# Towards synthetic catechol rich protein analogues through tyrosinase catalyzed activation of a tyrosine dipeptide in continuous mode

**DOI:** 10.1039/d5cy00361j

**Published:** 2025-10-22

**Authors:** Stefan Reinicke, Verena Jentzen, Felix Panis, Matthias Pretzler, Keven Walter, Ulrich Glebe, Annette Rompel

**Affiliations:** a Life Science & Bioprocesses, Fraunhofer Institute for Applied Polymer Research, IAP Geiselbergstraße 69 14476 Potsdam Germany Stefan.Reinicke@iap.fraunhofer.de; b Universität Wien, Fakultät für Chemie, Institut für Biophysikalische Chemie Josef-Holaubek-Platz 2 1090 Wien Austria https://www.bpc.univie.ac.at; c Laboratory for Organic Synthesis of Functional Systems, Department of Chemistry, Humboldt-Universität zu Berlin Brook-Taylor-Straße 2 12489 Berlin Germany; d Institute of Chemistry, University of Potsdam Karl-Liebknecht-Straße 24-25 14476 Potsdam Germany

## Abstract

We present a perspective towards a green synthesis route for synthetic, catechol rich protein analogues (TCC). The method relies on the oxidation of a tyrosine dipeptide in continuous mode by the immobilized tyrosinase *Sin*ATyr followed by Michael addition of a dithiol. For the dipeptide substrate a *k*_cat_ value of 0.16 s^−1^ and a *K*_m_ value of 1.6 mM were determined meaning that its conversion is slower and the affinity towards the active center of the enzyme is lower compared to the standard substrate l-tyrosine (*k*_cat_ = 5.6 s^−1^; *K*_m_ = 0.24 mM). For the continuous operation mode *Sin*ATyr is immobilized on polyelectrolyte decorated silica microparticles with a *k* value of 0.11 s^−1^ (at 1 mM dipeptide substrate) after immobilization and finally experimental proof is given that the converted dipeptide in contact with the dithiol yields the desired TCC structures.

## Abstract 2

We present a perspective towards a green synthesis route for synthetic, catechol rich protein analogues (TCC) based *via* a biocatalytic activation of tyrosine dipeptides in continuous mode.

## Graphical abstract

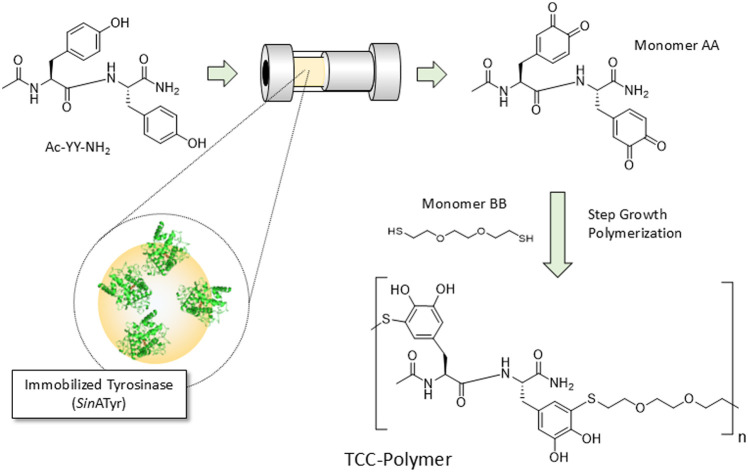

The utilization of enzymes as powerful, bio-based catalysts is well established nowadays. Enzymatic catalysis is also becoming increasingly important in organic synthesis, where for a long time only a few processes such as the hydrolysis of penicillin were used for commercial production.^1–3^ To a large extent, this success story originates from the use of optimized enzyme variants that work reliably under industrially relevant conditions. An example are tyrosinases, which are monophenoloxidases that are present in almost every living organism^4^ and that can be used as catalysts for environmentally friendly and efficient oxygenation of phenolic and catecholic structures (Scheme S1).^5^ Their utilization avoids the use of hazardous oxidation agents like sodium periodate or Fremy's salt.

The particular benefits stemming from the utilization of tyrosinases also open perspectives towards the generation of novel functional materials in polymer science. Arias *et al.* have been using the enzyme to transfer peptides containing a tyrosine and a cysteine residue into artificial glue proteins.^6^ Here, tyrosinase oxidizes the tyrosine motif into an *o*-quinone which subsequently reacts with the thiol groups of the cysteine in a Michael-type addition leading to polymerization (Scheme S1). The resulting TCC (thiol-catechol-connectivity) polymers can be seen as synthetic analogues to natural, Dopa-rich protein adhesives ubiquitous in mussels. In contrast to the latter, TCC polymers are not prone to spontaneous crosslinking, thus being much easier to handle while maintaining the excellent glue properties. In order to enhance structural diversity and gain more control over the material properties, the monomer base can be changed from the tyrosine/cysteine peptide, which represents a single, AB type monomer to an AA/BB system, in which an activated tyrosine dipeptide (AA) in combination with an independently variable dithiol (BB) is used. The dityrosine is supposed to get oxidized into the diquinone form by the tyrosinase and would then be exposed to the dithiol to form polymeric material in a step growth fashion (Scheme 1). The intrinsic problem with the monomer activation, however, is the necessity for oxidizing two tyrosine moieties per molecule. The half-oxidized (catechol-quinone) intermediate is capable of undergoing an intramolecular dimerization and is thus not available for follow up conversion into TCC species. This challenge can be addressed by implementing a flow process in which the activation is done either electrochemically^7^ or the enzyme is locally concentrated in an immobilized form with the substrate solution simply passing by. Immediately after escaping the flow reactor, the diquinone containing permeate can then be exposed to the dithiol leading to formation of TCC polymer. By that, unwanted side reactions are suppressed. Note that the flow process provides additional advantages over a conventional batch approach.^8–12^ The continuous nature of the process avoids product accumulation which in turn prevents unwanted enzyme deactivation. Additionally, the enzyme shelf life is increased and the workup procedure is kept simple (no biocatalyst has to be removed from the reaction mixture).

**Scheme 1:**
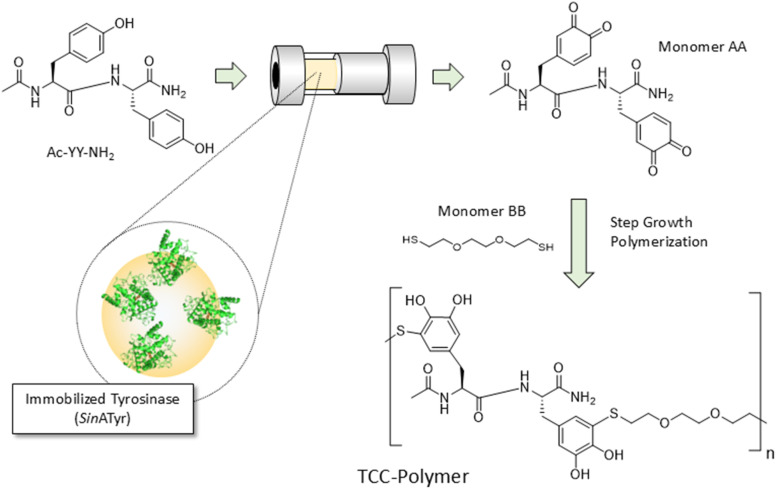
Depiction of the envisioned concept for the enzymatically backed synthesis of TCC polymers in a flow process.

Here, we suggest an immobilization scheme for a tyrosinase and provide a proof-of-principle for the described synthetic protocol for a new AA/BB-type TCC-species.

The biocatalyst of choice is the bacterial tyrosinase *Sin*ATyr, as it can be produced in larger quantities than the high-performance fungal tyrosinase used so far,^13^ while showing better activity towards *N*-acetyl-di-l-tyrosineamide (Ac-YY-NH_2_) in a preliminary test (not shown). On top of that the enzyme is expressed in its active form.^14^ The expression and purification protocol is described in the SI including an SDS-PAGE image of the expressed enzyme (Fig. S1).

Since *Sin*ATyr had not been exposed to Ac-YY-NH_2_ (Scheme 1) before, the first measure was to investigate its respective performance against this substrate. The catalytic parameters (*K*_m_ and *k*_cat_ values) for the oxidation of Ac-YY-NH_2_ by *Sin*ATyr in 50 mM sodium citrate buffer (pH 6.8) have been determined. (Fig. 1 and S3). We calculated a *K*_m_ value of 1.6 mM (±0.37 mM) and a *k*_cat_ value of 0.16 s^−1^ (±0.026 s^−1^). The catalytic behavior of *Sin*ATyr towards standard substrates such as l-tyrosine (*K*_m_ = 5.6 mM ± 0.30 mM, *k*_cat_ = 0.24 s^−1^ ± 0.047 s^−1^) and the corresponding catechol l-DOPA (*K*_m_ = 0.11 mM ± 0.028 mM, *k*_cat_ = 22 s^−1^ ± 1.5 s^−1^) has been investigated previously.^14^l-Tyrosine is a monophenol and represents the basic building block of the dimer Ac-YY-NH_2_ while l-DOPA is the corresponding catechol. Both standard substrates (l-tyrosine and l-DOPA) show higher *k*_cat_ values (higher activity), and lower *K*_m_ values (higher affinity), compared to Ac-YY-NH_2_. The lower catalytic activity of *Sin*ATyr towards Ac-YY-NH_2_, compared to standard substrates, can be explained by the comparatively bulky structure of Ac-YY-NH_2_ itself. Sterically large substrates cannot access the active center as effectively as smaller substrates, which reduces the catalytic activity of the enzyme-substrate combination.^14^

**Fig. 1:**
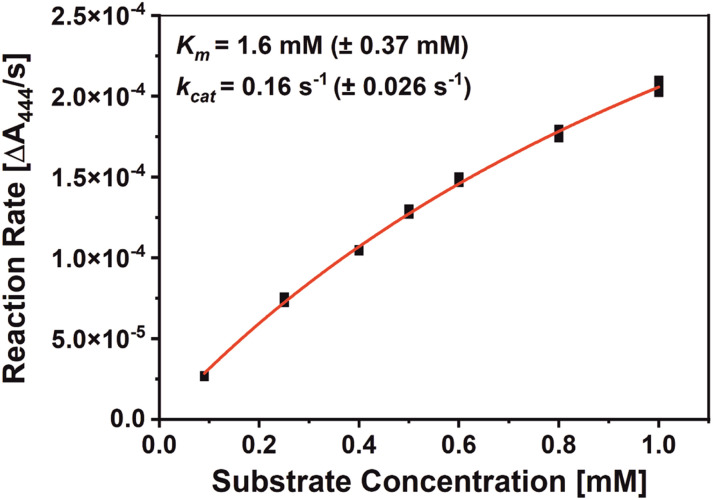
Michaelis–Menten plot of *Sin*ATyr on Ac-YY-NH_2_. Different Ac-YY-NH_2_ molarities were oxidized by *Sin*ATyr and the corresponding absorption values have been measured in triplicates in 50 mM sodium citrate buffer (pH 6.8).

As a necessary prerequisite for the flow process, the immobilization of tyrosinase is the next step to be carried out. Immobilization of enzymes is a long established strategy to enhance their performance or to optimize enzyme backed processes.^15,16^ In consequence literature protocols rely on many different carrier materials and bonding principles, even for tyrosinases alone. Most reported protocols, however, rely on silica particles^17^ and electrostatic (physical) bonding.^18–21^ Since micrometer sized silica particles are commonly used as column packing material and are generally favored in industry over other, softer carrier particles from a filterability standpoint, we decided to also rely on silica particles. A drawback, however, is the hard surface which may disturb the structure of the enzyme to an intolerable extent when large pressures are applied during the flow process. Therefore, we first decorated the particles with a polyelectrolyte, which in turn may then accommodate the enzyme. It has been shown before, that polycations like polyethyleneimine (PEI) or polydimethylaminoethylacrylate firmly stick to a silica surface without the need for covalent fixation which we could indeed easily verify by thermogravimetric analysis (Fig. S2). We noticed earlier when performing experiments on the tyrosinase *Ab*PPO4, however, that PEI-decorated particles were able to immobilize a rather high amount of enzyme from an enzyme containing buffer solution at pH 8, but subsequent washing steps with a buffer at lower pH (pH 6.8; working buffer for the actual biocatalytic process) revealed that a substantial amount of enzyme is released again. Thus, we needed to introduce additional functional groups to the polymer layer that fixate the enzyme by a few covalent bonds. γ-Butyrothiolactone (Tla) is a cyclic thioester, which reacts with the amino groups on the surface of proteins (Scheme 2).^22^ It can be introduced into polyacrylates or polyacrylamides *via* RAFT polymerization^23^ and has been used by us for enzyme immobilization purposes before.^24,25^ The polymer base for the immobilization was therefore constituted by poly(dimethylaminoethylacrylate-*co*-thiolactone-*N*-acrylamide) (P(DMAEA-*co*-TlaAm)). *Sin*ATyr is accommodated by electrostatic interaction *via* the positively charged DMAEA units and a few additional covalent bonds that are created by the reaction of the proteinogenic lysines with the TlaAm units.^24,25^ By that enzyme leaching should be largely prevented.

**Scheme 2:**
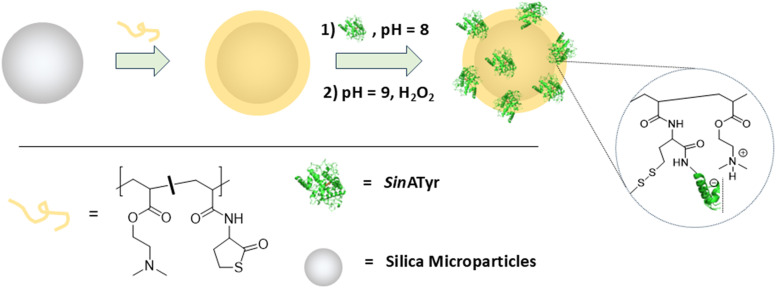
Protocol for the immobilization of *Sin*ATyr on polycation decorated silica microparticles *via* combined electrostatic and covalent binding.

At the beginning, we performed a basic immobilization parameter screening in order to find useful immobilization conditions (Fig. S4). It is obvious that smaller particles with their much larger available surface can in principle adsorb a relatively higher amount of enzyme. However, the handling of the particles becomes more delicate when their size decreases and the expected pressure drops in later fixed-bed reactors can be unbearably high. We chose commercially available particles with an average diameter of 16–24 μm or 40–75 μm. The Tla-content of the enzyme accommodating polymer layer was varied between 17 and 20 mol% per polymer chain. In this range, both a good immobilization yield as well as sufficient covalent fixation is ensured. When loading the particles with polymer prior to the actual enzyme immobilization step, the concentration of polymer in the dispersion was limited to 0.2% (m/v). Higher values lead to a drastically reduced immobilization yield later on as well as to a slightly reduced operational stability. It is assumed that the stability of the polymer layer itself is compromised when its thickness exceeds a certain value. The actual immobilization is performed in buffered solution at pH 8, followed by a post immobilization treatment where the pH is raised to 9 and a small amount of hydrogen peroxide is added. These post treatment conditions further boost hydrolysis/aminolysis of the Tla groups and promote disulfide formation for further stabilization of the enzyme accommodating matrix.^24,25^ We performed an additional leaking test under the conditions later established for the operation of the biocatalytically active particles in flow mode. There is a slightly enhanced absorption detected in the permeate at 280 nm compared to the plain buffer which at a first glance points to leaked enzyme. However, the same appears when plain silica particles are used instead of enzyme loaded species, meaning that the apparent absorption signal stems in fact from a small fraction of permeated particles that scatter away part of the incident light (Fig. S5). Thus, no detectable amount of enzyme is leaking.

The enzyme loaded particles were first used in a simple batch process from which samples were taken at different times which in turn were subjected to HPLC analysis. Here, we used the enzyme immobilized on the larger particles in order to be able to completely remove all solid material upon centrifugation before the samples get injected into the HPLC device. A clear picture evolves (Fig. 2) showing how Ac-YY-NH_2_ (structure c) is getting converted into the respective monoquinone (monoactivation, structure b) and into the diquinone (bis activation, structure a). However, while the Ac-YY-NH_2_ trace completely vanishes in the chromatogram over time, so do the traces of the reaction products, pointing out the limited stability of both the monooxidation product and the diquinone. The monitoring of the absorption spectrum of the reaction product over time indicates a complete vanishing of the formed absorption band over the course of 1 hour (Fig. S3). At this point the envisioned flow process comes into play. As the substrate solution is immediately transported away after contact with the immobilized enzyme and brought into contact with the dithiol 2,2′-(ethylenedioxy)diethanethiol, it can be converted to the desired product before an adverse side reaction takes place on a significant scale.

**Fig. 2:**
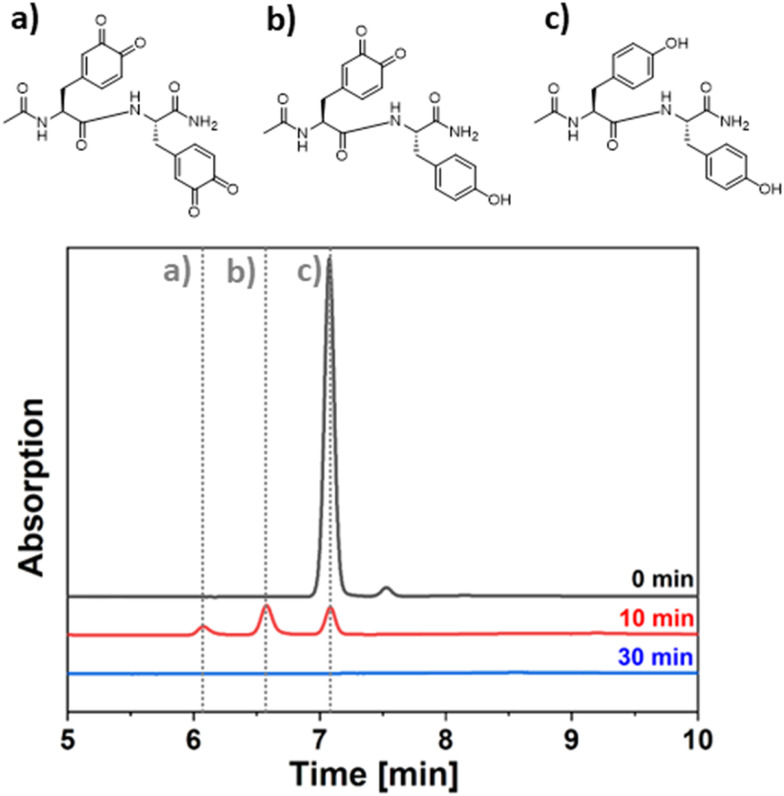
HPLC chromatograms for samples extracted from the reaction mixture for the conversion of Ac-YY-NH_2_ by immobilized *Sin*ATyr in batch mode. The chromatogram was recorded using a reversed phase column (C18) running a gradient of water/acetonitrile (+0.05%(v/v) formic acid) from 95/5 v/v to 5/95 v/v over the course of 17 min. Detection was done *via* UV absorption at 270 nm.

In order to assess the impact of the immobilization on the apparent specific enzyme activity immobilization yields were determined (Table S1). This was done indirectly by measuring the residual protein concentration in the supernatant *via* BCA assay after removal of the enzyme loaded particles. The immobilization batch, where larger particles (diameter 40–75 μm) have been used, carries 0.7 μg enzyme per mg particle. These particles were then used in flow mode to assess their activity (Fig. 3). A substrate solution with a concentration of 1 mM was pumped through the syringe filter containing the particles and 5 fractions à 0.5 mL were collected with a flow rate of 50 μL min^−1^. Please note that the low conversions detected are the result of the low amount of immobilized enzyme applied in this experiment, which, however, was sufficient for the assessment of enzymatic activity and operational stability. For the first collected fraction, a higher conversion is reached (7% monoquinone in the chromatogram), which then drops by a significant extent but stays stable for the remainder of the experiment (∼4% monoquinone). We believe that this is due to irreversible adsorption of a small fraction of product on the particles (visible by the naked eye) which changes the environment of the enzyme to some extent before a stable process is reached. With 11.4 μg immobilized enzyme used in the experiment, the results translate into a *k* value of 0.11 s^−1^ (see experiment description, SI page 10). The *k*_cat_-value for the non-immobilized enzyme is 0.16 s^−1^, which corresponds to a *k* value of 0.062 s^−1^ at a substrate concentration of 1 mM. If the experimental *k* value under flow conditions, which corresponds to monoquinone formation, is halved to compare it with *k*_cat_ (which refers to diquinone formation) the resulting 0.053 s^−1^ correspond to 87% of the activity exhibited by the free enzyme. The activity loss upon immobilization is thus only very moderate.

**Fig. 3:**
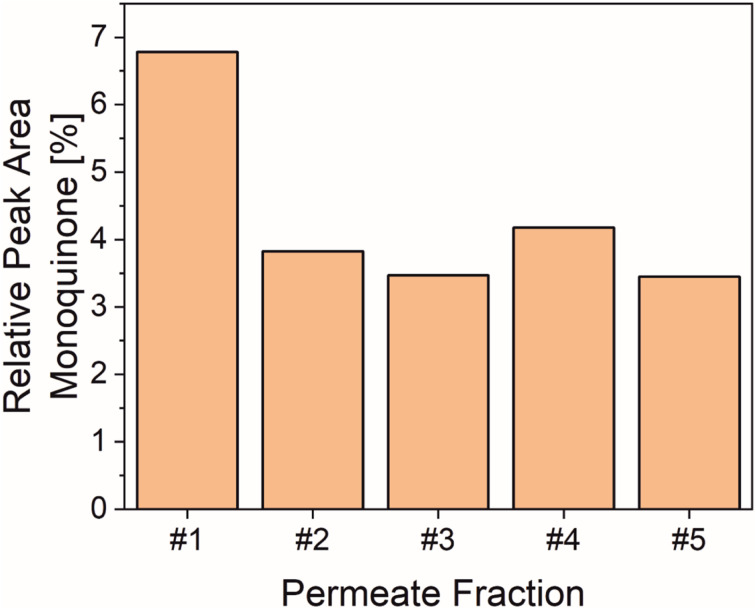
Extracted HPLC data for the conversion of dityrosine by immobilized *Sin*ATyr in flow mode. The peak areas for the substrate dityrosine and the monooxidation product (= monoquinone) within the respective chromatogram for each of the collected permeate fractions were detected and summed up. The peak area portion for the monoquinone with respect to this sum is then plotted (“Relative Peak Area Monquinone”). Each fraction had a volume of 500 μL and the flow rate was set to 50 μL min^−1^. The amount of immobilized enzyme was 11.4 μg and substrate concentration was set to 1 mM (50 mM sodium citrate buffer, pH 6.8).

In order to provide a final proof-of-principle of our proposed synthetic concept for the TCC material, another batch of biocatalytically active particles was collected within a syringe filter followed by controlled pumping of substrate solution through this filter. This time, smaller particles (diameter 16–24 μm) were used with a slightly lower amount of thiolactone units in the polymer decorating the particles. This ensures a maximum enzyme loading (2.3 μg of enzyme per mg particle). The permeate was allowed to drop immediately into a dithiol containing buffer solution. Even with the higher amount of applied enzyme, it is likely that the conversion of the dityrosine is still incomplete. Additionally, since it requires some effort to match the concentration of the two monomers (diquinone as monomer AA and dithiol as monomer BB) to an extent that is necessary for a substantial molecular weight build (step growth polymerization: Carothers' equation), it seemed the best option at this stage to use dityrosine in excess compared to the dithiol (monomer BB) but let it drop slowly into the thiol containing vessel. By that we should eventually reach the stochiometric monomer ratio which is required for a proper molecular weight build up in an AA/BB type step growth polymerization. The weight limiting influence of chain stopping monoquinone, however, can only be mitigated by optimization of the biocatalytic activation step itself. This issue still needs to be addressed in a follow-up work.

In the batch process, the substrate solution initially adopted a dark red to violet color. Now, in the flow process after the contact with the dithiol, the solution appeared light brown to orange, indicating the formation of the desired TCC structures. The final product was subjected to molar mass analysis (Fig. 4). The MALDI-ToF mass spectrum mainly shows the AA-BB-AA species and a peak of lower intensity indicating an AA-BB-BB-AA structure (stemming from dimerization of BB through disulfide formation prior to the Michael addition to AA). AA, for both species, is constituted mainly of the monoquinone highlighting the ability of the latter to terminate the growing chains. There are also peaks detectable at 1017 and 1199 *m*/*z* respectively (marked by asterisk in Fig. 4) which point to the presence of AA–BB–AA structures in which one monoquinone is replaced by diquinone (1017 and 1199 *m*/*z*). These peaks, however, are only present in low intensity. This means that the product mixture from the enzymatic activation is still largely dominated by the monoquinone. Nevertheless, the results show that the activated AA species reacted with the dithiol in the anticipated manner, thus proofing the principal working of our concept.

**Fig. 4:**
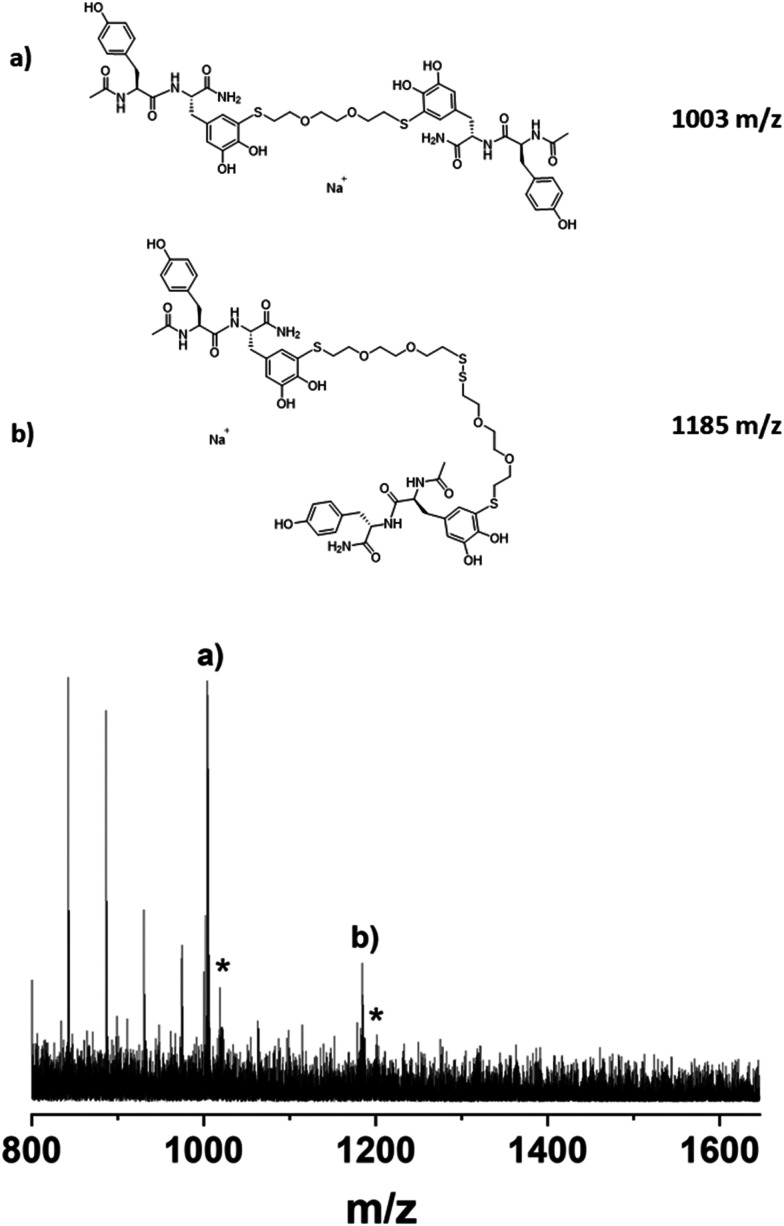
MALDI-ToF analysis after enzymatic activation of Ac-YY-NH_2_ in flow mode and subsequent exposure to 2,2′-(ethylenedioxy)diethanethiol. The Ac-YY-NH_2_/dithiol ratio had been set to 3.3. The matrix for the MALDI-ToF measurement was dithranol.

In conclusion, we showed how a tyrosinase can be utilized to activate dityrosine to generate TCC-based adhesive protein analogues in a flow process and demonstrated a basic proof-of-principle. Optimization of the process will be the next necessary step. The performed kinetic analysis with respect to the substrate Ac-YY-NH_2_ will help to generate new, more efficient enzyme variants, while a first scale up should be tackled to provide enough enzymatically active material to pack and run lab scale preparative columns for the monomer activation. This will allow a detailed study of the process itself with the ultimate aim to achieve complete dityrosine activation as a necessary prerequisite for the generation of TCC-typical material that goes beyond an oligomeric nature.

## Conflicts of interest

There are no conflicts to declare.

## Supplementary Material

CY-015-D5CY00361J-s001

## Data Availability

The data supporting this article have been included as part of the supplementary information (SI). Supplementary information: containing detailed experimental protocols of all syntheses and analyses, as well as supplemental data on enzyme structure and biocatalytic performance and the enzyme immobilization. See DOI: https://doi.org/10.1039/d5cy00361j.
