# Supplementary material for: Towards synthetic catechol rich protein analogues through tyrosinase catalyzed activation of a tyrosine dipeptide in continuous mode
Source: Catal Sci Technol. 2025 Oct 22;15(22):6660–5. doi: 10.1039/d5cy00361j (PMC12573765; doi:10.1039/d5cy00361j)
Supplement: CY-015-D5CY00361J-s001 [file CY-015-D5CY00361J-s001.pdf]

Supplementary Information (SI) for Catalysis Science & Technology.
This journal is © The Royal Society of Chemistry 2025

Towards synthetic catechol rich protein analogues through tyrosinase catalyzed activation of dityrosine in continuous mode

Stefan Reinicke*,a), Verena Jentzen[a)], Felix Panis[b)], Matthias Pretzler[b)], Keven Walter[c)], Ulrich Glebe[d)], Annette Rompel[b)]

a) *Life Science & Bioprocesses, Fraunhofer Institute for Applied Polymer Research, IAP, Geiselbergstraße 69, 14476 Potsdam, Germany*
b) *Universität Wien, Fakultät für Chemie, Institut für Biophysikalische Chemie, Josef-Holaubek-Platz 2, 1090 Vienna, Austria; www.bpc.univie.ac.at*
c) *Laboratory for Organic Synthesis of Functional Systems, Department of Chemistry, Humboldt University Berlin, Brook-Taylor-Straße 2, 12489 Berlin, Germany*
d) *Institute of Chemistry, University of Potsdam, Karl-Liebknecht-Straße 24-25, 14476 Potsdam, Germany*

Materials & Methods / Supplemental Data

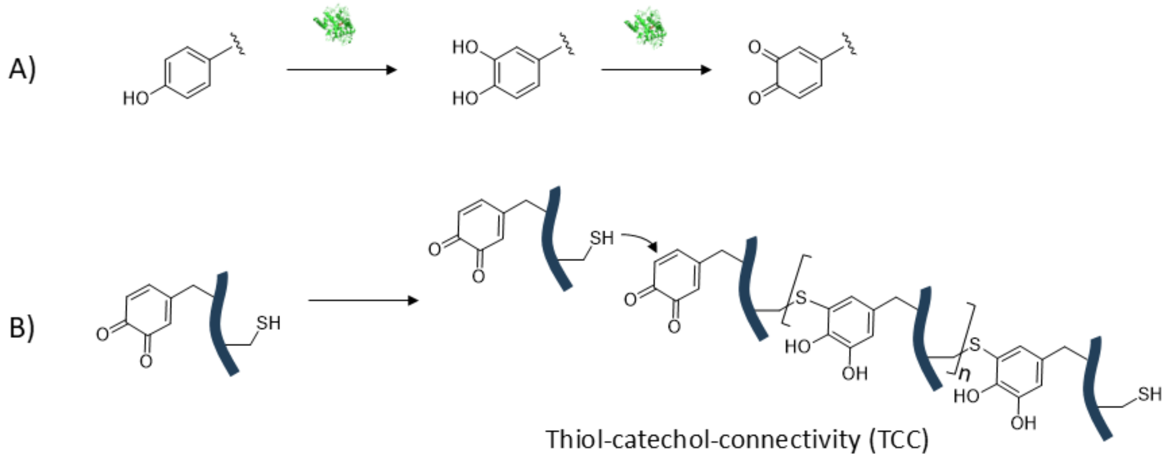

Scheme S1. A) Schematic depiction of the two-step oxidation of phenolic moieties by tyrosinases to yield the respective quinone, B) quinone and thiol containing peptide undergoing an intramolecular Michael-type addition yielding TCC type polymers.[1]

Recombinant expression and purification of *Sin*ATYR. (UniProt ID: L0D705, MW: 40650.0 Da)

The sequence-verified expression vector containing the gene coding for *Sin*ATYR with a 10xHis tag placed upstream of the TYR gene was transformed into chemically competent cells (*E. coli* BL21(DE3)). Transformed cells were then spread on an agar plate containing 100 µg/mL ampicillin and incubated for 16 h at 37 °C to allow for colony formation. Single colonies were picked and used to inoculate 400 mL of autoclaved LB medium containing 5 *g*/l yeast

extract, 10 *g*/l tryptone, 10 *g*/l NaCl, as well as 100 µ*g*/mL ampicillin. The expression medium was placed in a shaker-incubator at 240 rpm and 37 °C until the OD_{600} reached a value of 0.9−1.2, which is a rather high value. It is noted, though, that tyrosinases, especially in their active form (as opposed to latent tyrosinases that feature a lid-like domain that covers their active site), generate significant biochemical stress to the producing cell The bacterial genus Streptomyces produces its tyrosinases in a way that ensures activation happens outside of the cell[2] and some of its members even feature a class of tyrosinases thought to be used mainly for suppressing competing organisms.[3] With this in mind, induction at a later growth stage can be advantageous for the production of heterologous tyrosinases as a favorable tradeoff between the total amount of produced enzyme (dramatically up) versus degree of solubility (slightly to markedly down, but sometimes even up). To start the expression of *Sin*ATYR, 0.5 mM $CuCl_2$ and 0.5 mM isopropyl-β-D-thiogalactopyranoside (IPTG) were added to the expression culture. Protein expression was performed for 96 h at 10 °C. Thereafter, the expression culture was centrifuged at 3500 g for 10 min at 4 °C before the cell pellet was resuspended in 40 mL cell lysis buffer (10 mM imidazole, 50 mM NaCl, 50 mM TRIS, pH 9.0, 4 °C). The cell lysis was performed in the cell lysis buffer by freezing the sample in liquid N_2, followed by thawing in a 25 °C water bath for four times. 7 mM $MgCl_2$ and 0.03 *g*/l DNase I (Sigma-Aldrich, Vienna, Austria) were added to the resulting highly viscous cell lysate and the mixture was incubated at 4 °C for 15 minutes. Next, the samples were centrifuged at 25,000 g at 4 °C for 20 minutes and filtered (PES 0.45 µm) before they were applied to a HisTrap HP 5 mL column (GE Healthcare, Freiburg, Germany) using an ÄKTA purifier system placed in a refrigerator at 4 °C. Samples were washed using 10 column volumes cell lysis buffer (4 °C) and eluted using cell lysis buffer supplemented with 400 mM imidazole (4 °C). After the elution process, the buffer was exchanged to a storage buffer (50 mM NaCl, 50 mM TRIS, pH 9.0, 4 °C) using a Vivaspin ultrafiltration device (VWR, molecular weight cut-off 30 kDa).

Purified *Sin*ATYR was quantified *via* the Lambert-Beer law by measuring the absorption of the protein sample at 280 nm using extinction coefficients (2.070 $(g/l)^{-1}$) calculated by the ExPASy ProtParam tool[1]. The purity of the protein was determined by a reducing 12.5% SDS-PAGE (Figure S1).

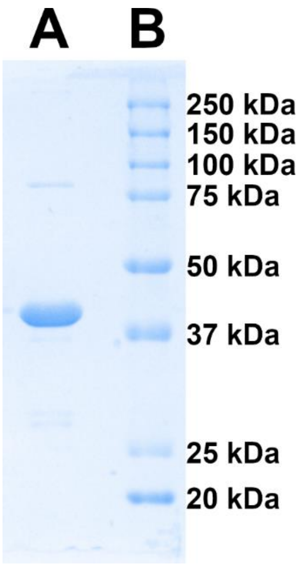

Figure S1. SDS-PAGE of recombinantly expressed *Sin*ATYR. A = 10 µg *Sin*ATYR, B = Precision Plus Protein Dual Color Standards protein marker from Bio-Rad. The Figure has been created using GIMP 2.10.18 (https://www.gimp.org). The full length of the polyacrylamide gel is shown in Figure S1.

Synthesis of *N*-Acetyl-di-L-tyrosineamide (AC-YY-NH_2)

The synthesis of AC-YY-NH_2 was achieved via solid-phase peptide synthesis (SPPS) using am Fmoc-based procedure. A rink amide resin (loading 0.74 mmol/*g*, Iris Biotech) was used as solid phase with 15 mmol as batch size. The resin was placed in a solid-phase reactor. For 1 *g* of resin 15 mL of solvent was used. Between each step (coupling, deprotection, capping) the resin was washed 5 times with *N*-methypyrrolidon (NMP, peptide grade, Iris Biotech), 5 times with dichloromethane (DCM) and again 5 times with NMP.

First, the resin was allowed to swell in a NMP/DCM mixture (1/1, v/v) for 1 h at room temperature. After washing with NMP 10 times, the resin was mixed with deprotection solution (20 vol% piperidine in NMP) (piperidine: peptide grade, Iris Biotech) and shaken at room temperature. For the first coupling step, the deprotected, swollen and washed resin was mixed with a solution of 5 eq. Fmoc-L-Tyr(tBu)-OH (Iris Biotech), 5 eq. Benzotriazol-1-yloxytripyrrolidinophosphonium hexafluorophosphate (PyBOP, Carl Roth) and 5 eq. *N,N*-Diisopropylethylamine (DIPEA, Iris Biotech) in NMP. The reaction mixture was shaken at room temperature for 5 min. After another washing step, deprotection and coupling was repeated, this time 10 eq. of DIPEA. Final deprotection and capping (capping solution: 9.5 mL acetic anhydride, 4.5 mL DIPEA, 0.4 *g* HOBt in 200 mL NMP) was performed as described above.

Prior to the splitting of the dipeptide from the resin, the latter was washed with NMP and DCM and dried for 20 min under reduced pressure. The resin was then shaken with the splitting

solution (TFA/water/triethylsilane 95/2.5/2.5, v/v/v) (TFA: Iris Biotech) for 2.5 h. The liquid was then partially removed in an argon gas stream and the remaining solution was poured into cold diethyl ether for precipitation of the product. The precipitate was isolated from the supernatant via centrifugation and dried under reduced pressure.

Final purification was performed via preparative HPLC using a reversed phase column with eluent gradient of water: Acetonitrile (+ 0.1 % formic acid) (water: purified on SG LaboStar TM 1-UV system; acetonitrile: LC-MS grade, VWR International; formic acid: 99 %, Acros Organics) starting from 90:10 to 10:90 over the course of 22 min.

Synthesis of poly(dimethylaminoethyl acrylate-*co*-thiolactone-N-acrylamide) (PDMAEA-*co*-TlaAm)

P(DMAEA-*co*-TlaAm) was synthesized via RAFT polymerization. The synthesis of the comonomer TlaAm is described elsewhere.[4] For a typical polymerization a reaction mixture composed of DMAEA (> 98.0 %, TCI) passed through a short column of activated neutral alumina (Merck) first, TlaAM, 2-(dodecylthiocarbonothioylthio)-2-methyl propionic acid (DDMAT, 98 %, Sigma-Aldrich) and AIBN (98 %, Sigma-Aldrich, recrystallized from $CHCl_3$) with a ratio of 80:20:1:0.1 and an overall monomer concentration of 1.3 mol/L in 4 mL non-stabilized dioxane (≥ 99.8 %, Carl Roth) was degassed by repeated freeze-pump-thaw cycles and then placed in an oil bath set to 80 °C for 5 h. After that the reaction solution was first cooled down in an ice/water bath and then exposed to air. The volume of the reaction solution was doubled by adding sodium citrate buffer (50 mM, pH = 6.8). The mixture was then placed in a dialysis tube (SpectraPor, regenerated cellulose, MWCO = 3.5kD) and dialysed first against the same buffer and then against MilliQ-water. The aqueous solution of the polymer was then subjected to freeze drying yielding the final product.

Synthesis of (PDMAEA-*co*-TlaAm)-decorated silica microparticles

In a typical procedure, 200 mg silica particles (Ø = 16-24 µm, Carl Roth) were weighed into a 50 mL falcon tube followed by addition of 10 mL MilliQ-water. Parallel to that, 30 mg of P(DMAEA-*co*-TlaAm) were dissolved in 5 mL MilliQ-water. Polymer solution and particle dispersion were then mixed and shaken for 1 h at room temperature. After that, the particles were washed 6 times with MilliQ-water, centrifuged and dried in vacuo over night at 40 °C. Stability against the operating buffer (sodium citrate, 50 mM, pH 6.8) was assessed by running thermogravimetric analysis (TGA2, Mettler Toledo, 10 K/min up to 600 °C) on the particles before and after thorough shaking in the buffer (Figure S2).

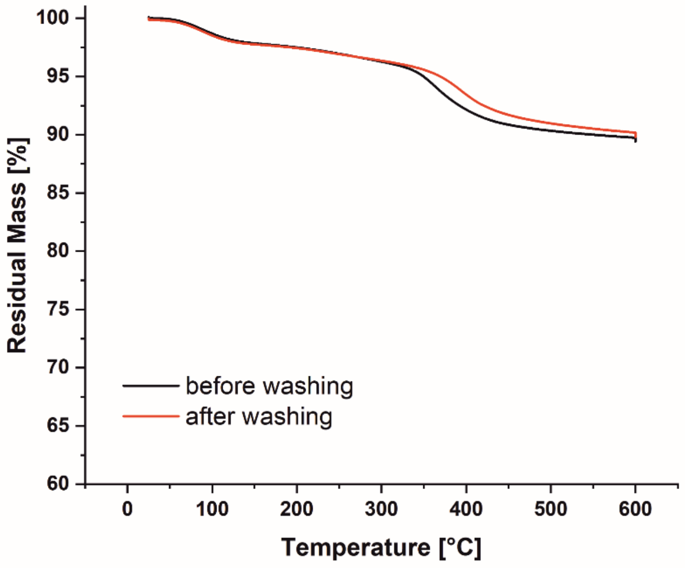

Figure S2. Thermogravimetric analysis of the P(DMAEA-*co*-TlaAm) decorated silica microparticles (diameter 16-24 µm) before and after washing with the operating buffer for the biocatalysis (sodium citrate, 50 mM, pH 6.8).

Investigation of the catalytic activity of *Sin*ATYR towards Ac-YY-NH_2.

To identify an absorption wavelength suitable for kinetic investigations, Ac-YY-NH_2 (100 µM) was oxidized using an excess of *Sin*ATYR (1 mg) in 50 mM sodium citrate buffer (pH 6.8) in a total volume of 1 mL for 30 minutes until the reaction has been completed. Then, absorption spectra were recorded from 250 – 800 nm to analyze the stability of the formed reaction product. An isosbestic point was identified at 444 nm, which has been used for further kinetic measurements (Figure S3). Measurements were performed on a Shimadzu UV-1800 spectrophotometer at 25 °C.

Next, the molar extinction coefficient of the reaction product at 444 nm was determined. Different molarities of Ac-YY-NH_2 (0.1 – 0.65 mM) were mixed with 50 µg *SinA*TYR in 50 mM sodium citrate buffer (pH 6.8) in a total reaction volume of 200 µl. After the reactions were completed, the absorption values were recorded and plotted using the OriginPro 8 software. The molar extinction coefficient was determined using a linear fit based and the least-square-method, which resulted in an extinction coefficient (at 444 nm) of (1311 ± 25) $M^{-1}cm^{-1}$ (Figure 1A, main manuscript). Measurements were performed on a TECAN infinity M200 photometer (Tecan, Salzburg, Austria) in 96-well plates in triplicates.

For the determination of the catalytic parameters K_m and k_{cat} for the oxidation of Ac-YY-NH_2 by *Sin*ATYR in 50 mM sodium citrate buffer (pH 6.8) 50 µg *Sin*ATYR were mixed with seven different substrate concentrations (0.1 – 1 mM) in 50 mM sodium citrate buffer (pH 6.8) in a total volume of 200 µl. Maximum reaction rates were determined.

A)

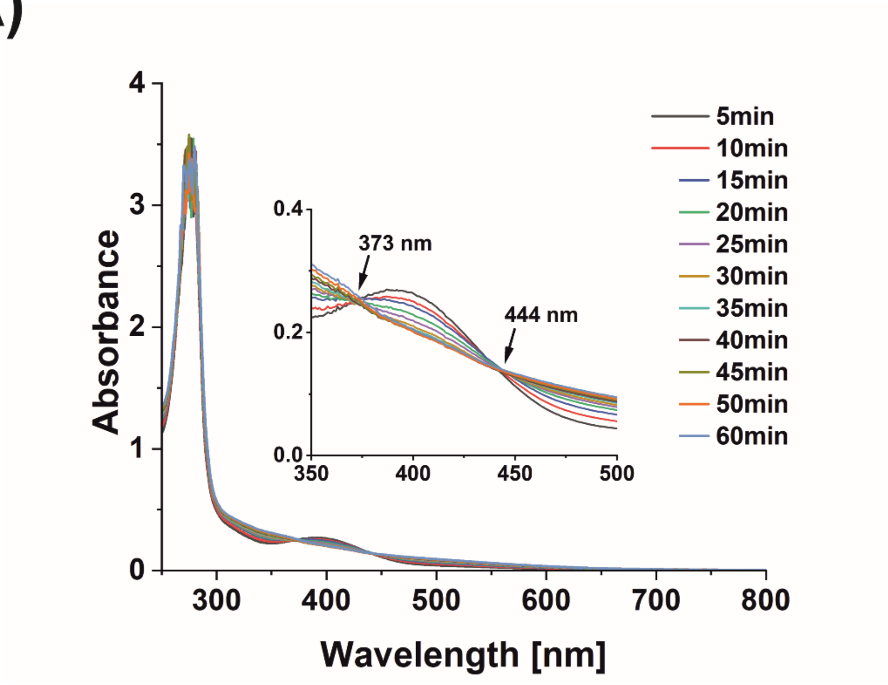

B)

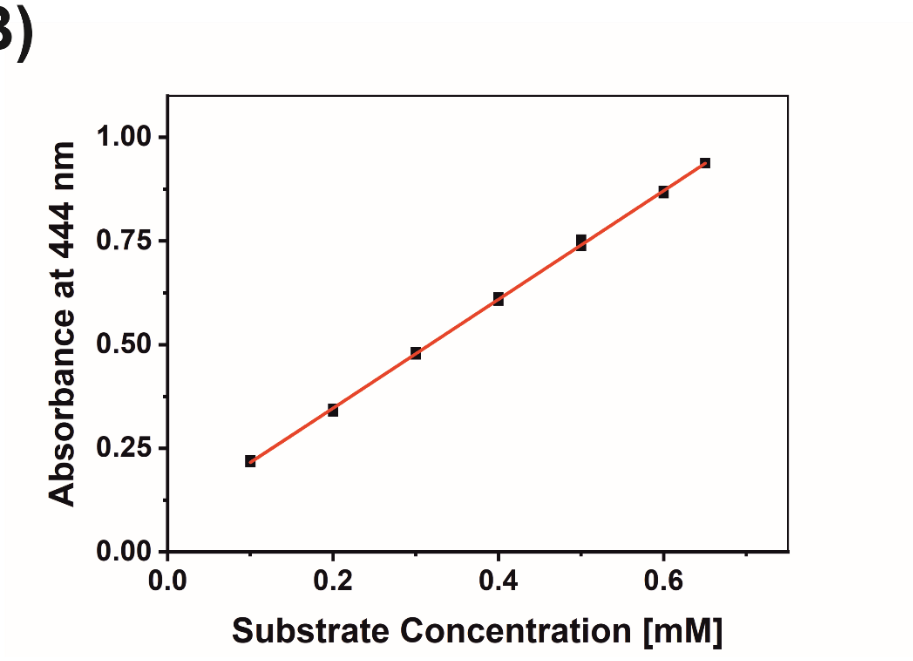

Figure S3. Absorption characteristics of the Ac-YY-NH_2 reaction product. A) Absorption spectra. The stability of the product formed by the oxidation of Ac-YY-NH_2 by *Sin*ATYR has been investigated over time in 50 mM sodium citrate buffer (pH 6.8), which identified an isosbestic point at 373 nm and at 444nm (see inlet). The isosbestic point at 444 nm has been used for further analysis as it appears more sharply, in contrast to the isosbestic point at 373 nm. **B) Absorbance measured at 444 nm as a function of substrate concentration**, including a linear regression line (red).

Immobilization of *Sin*ATYR

In a typical procedure, the *Sin*ATYR containing solution was diluted with sodium citrate buffer (50 mM, pH 8) to reach a concentration of 1 m*g*/mL. 100 mg of polymer decorated silica particles were mixed with 500 µL of the diluted *Sin*ATYR solution in an 1.5 mL Eppendorf vial. The latter was placed in a thermoshaker and shaken for 1 h at 20 °C with 900 U/min. The particles were centrifuged off and washed 6 x with MilliQ-water. The washed particles were then mixed with 500 µL diluted H_2O_2 (0.1 % in sodium citrate buffer, 50 mM, pH 9) and shaken for 10 min. Again, the particles were centrifuged off and washed 6 x with MilliQ-water. The supernatant of the last washing step was subjected to BCA assay analysis (ThermoScientific Micro BCA Assay Kit) to verify the absence of any enzyme leaking. As a final step water was added to the particles until 600 m*g* of dispersion was obtained. Relative enzyme loading and stability was estimated with a Panta Prometheus Nano-DSF device (Nanotemper), recording fluorescence intensities before and after shaking the enzyme loaded particles in the operating buffer (sodium citrate, 50 mM, pH 6.8) (Figure S4). Two different sets of immobilization parameters were later used for further experiments (Table S1). For these two batches exact immobilization yields were determined. This was done by determining the residual protein concentration in the supernatant after removal of the enzyme carrying particles. Here, absorption was measured by applying a BCA assay kit (Pierce™, ThermoScientific).

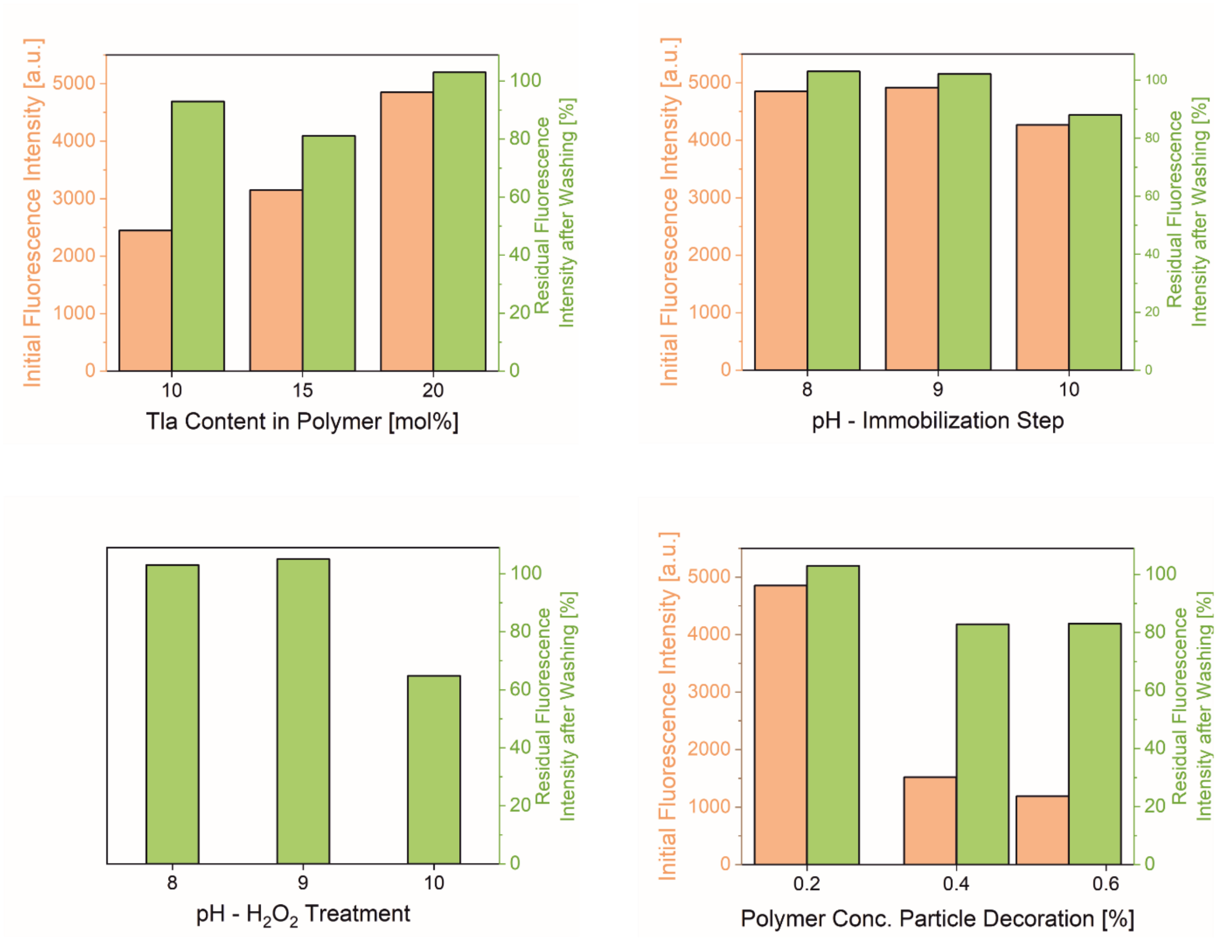

Figure S4. Parameter Screening for the optimization of the immobilization protocol of *Sin*ATYR on polycation decorated silica microparticles (see Scheme 2, main manuscript). We recorded fluorescence intensities as a measure for protein content using a NanoDSF device (Prometheus Panta, Nanotemper). "Initial Fluorescence Intensity" (orange columns) refers to the initial enzyme loading while "Residual Fluorescence Intensity after Washing" (green columns) is a measure of loading stability towards shaking in the operating pH6.8 sodium citrate buffer. Immobilization parameters in focus were the thiolactone content in the polymers with which the microparticles were decorated prior to immobilization ("Tla Content in Polymer"), the pH during the actual immobilization step ("pH – Immobilization Step"), the pH during the post-immobilization treatment with H_2O_2 ("pH – H_2O_2 treatment") and the polymer concentration during the decoration of the microparticles with polymer prior to the immobilization step ("Polymer Conc. Particle Decoration").

Table S1. Summary of the immobilization parameters and immobilization yields for the two immobilization batches used for the follow-up activity studies. For both batches the pH during the immobilization step was set to 8. The pH for the H_2O_2-treatment right after immobilization was set to 9. For both batches the concentration of polymer during the decoration of the particles with polymer prior to immobilization was set to 0.2 %.

Batch #	Tla content in particle decorating polymer [mol%]	Particle diameter [µm][a]	Immobilization yield [%][b]	Enzyme per particle [µg/mg]
1	20	40-75	14.2	0.7
2	17	16-24	46.8	2.3

a) Values given by the manufacturer
b) 100 mg polymer decorated particles dispersed in 0.5 mL enzyme solution (1 mg/mL *Sin*ATyr)

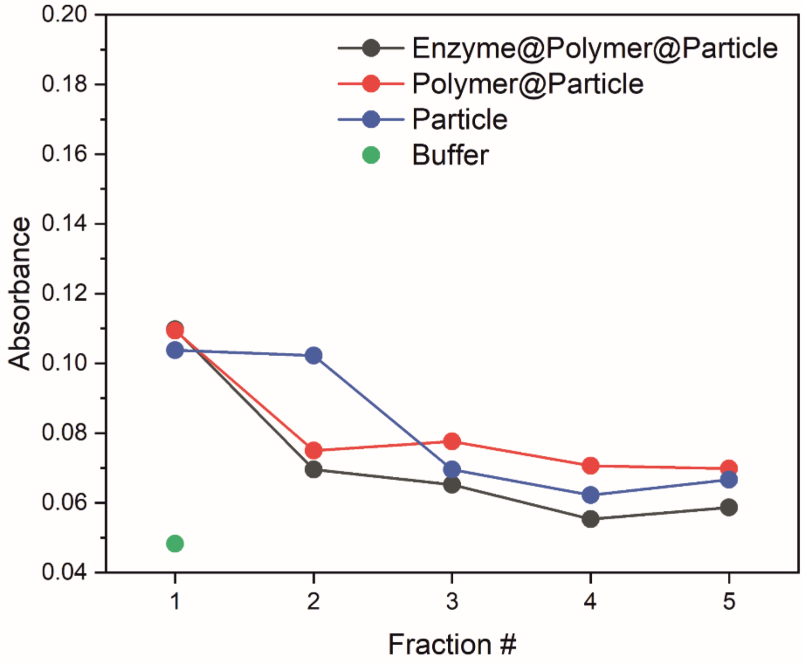

Figure S5. Enzyme leaking test for immobilized *Sin*ATyr applied in flow mode. Buffer was pumped through a syringe filter containing the respective particles and collected permeate fractions were subjected to photometric analysis: Absorptions were measured at 280 nm (ε_{280} of *Sin*ATyr: 83880 M^{-1} cm^{-1}). "Enzyme@Polymer@Particle" refers to the biocatalytically active particles that contain the enzyme. "Polymer@Particle" refers to the polymer decorated particles that are ready for enzyme immobilization. "Particle" refers to the plain silica particles that have not been modified. The experiment has been performed with the immobilization batch #1 (see Table S1)

Biocatalytic conversion of Ac-YY-NH_2 with immobilized *Sin*ATYR

Batch

The enzyme loaded particles (Batch #1, Table S1) were collected from the stored dispersion after centrifugation and decanting (dry mass 200 mg). 2.5 mL substrate solution (Ac-YY-NH_2 0.38 mg/mL in 50 mM sodium citrate buffer pH 6.8) were added and the entire mixture continuously vortexed over the course of the experiment. At selected times, samples were taken and rigorously centrifuged to remove any solid material. The collected liquid fractions were subjected to HPLC analysis.

Flow Mode

100 µL of enzyme loaded particles (batch #1, Table S1) was pushed through a syringe filter (Whatman, cellulose acetate, 0.2 µm). The dry mass of particles loaded into the filter was determined through the particle concentration in the dispersion in batch #1, which has been measured gravimetrically earlier on. Together with the determined enzyme immobilization yield, the total amount of enzyme used was 11.4 µg. 2.5 mL of substrate solution (1 mM of Ac-YY-NH_2 in 50 mM sodium citrate buffer, pH 6.8) was pumped through the filter at a constant flow rate of 50 µL/min. The permeate was collected and immediately being subjected to HPLC analysis. For this analysis, extinction coefficients for the substrate and the monooxidation product were assumed to be the same at the used detection wavelength. The specific enzyme activity has been calculated in the following way:

The free volume V_R of the particle-loaded syringe filter which acts as the flow reactor is 0.6 mL. With a flow rate of $\dot{V}$ = 50 µL/min, the dwell time τ can be determined:

$$\tau = \frac{V_R}{\dot{V}} = \frac{0.6\,mL}{0.05\,mL/min} = 12\,min = 720\,s$$

The average conversion of dityrosine for the permeate fractions 2-5 was 3.6 % (Figure 3, main manuscript; no diquinone could be detected in any of the fractions). The conversion rate is then calculated as follows:

$$v = \frac{c \cdot V_R \cdot 0.036}{\tau} = \frac{0.001\,M \cdot 0.0006\,L \cdot 0.036}{720\,s} = 3 \cdot 10^{-11}\,\frac{mol}{s}$$

With 11.4 µg of enzyme (0.28 nmol) used the turnover number k is then calculated as 0.11 s^{-1}.

For the second experiment in which the formation of TCC-structures was to be confirmed, a larger amount of a dispersion of enzyme loaded particles (batch #2, Table S1) was pushed through a syringe filter (Whatman, cellulose acetate, 0.2 µm) until no more material could be taken up. Drying and weighing after the flow through experiment revealed a particle mass of 336 mg accommodated within the filter. 20 mL of substrate solution (1 mM of Ac-YY-NH_2 in 50 mM sodium citrate buffer, pH 6.8) was pumped through the filter at a constant flow rate of 131 µL/min. The permeate was allowed to drop into a small vial containing 1 µL of 2,2'-(ethylenedioxy)diethanethiol in 0.5 mL sodium citrate buffer (50 mM, pH 6.8). The collected mixture was freeze dried, redissolved in 5 mL dimethylformamide (DMF) and filtered using a

syringe filter (Whatman, cellulose acetate, 0.2 µm) before being subjected to SEC and MALDI-ToF analysis.

High-performance liquid chromatography (HPLC)

Two different devices were used for HPLC analysis:

For the batch mode experiment a ThermoFisher Vanquish HPLC system (quaternary pump VF-P20-A, Split Sampler FT VF-A10-A, diode array detector FG VF-D11-A) using an inversed phase column (AppliChrom OUT LipoMare C18, 105 Å, 5 µ, 250 x 4 mm) was used. Elution was done by running a gradient at 0.7 mL/min and 40 °C over the course of 30 min involving water/acetonitrile (both containing 0.05 %(v/v) formic acid): 0-17 min from 95/5 to 5/95 v/v, from 17 to 23 min at 5/95 followed by immediate switch back to 95/5 for the remainder of the run. Detection was done by monitoring absorption at 270 nm. Identification of compounds was done with the aid of the connected ESI mass spectrometer (Vanquish ISQ EM, positive mode).

For the flow mode experiment an ACUIDITY-UPLC® H-Class CM Core System of Waters GmbH (Eschborn, Germany) was used. Detection was done utilizing an ACUIDITYUPLC® photo diode array (PDA)-detector (wavelength range 190-500 nm). Separation was conducted with ACUIDITY-UPLC® BEH C18 VanGuardTM precolumn (110 Å, 1.7 µm, 5 × 21 mm ID) and an ACUIDITY-UPLC® BEH C18-column (110 Å, 1.7 µm, 5×21 mm ID) from Waters. As mobile phase, mixtures of solvent A (Milli-Q water with 0.1% formic acid (FA), v/v) and solvent B (acetonitrile with 0.1% FA, v/v) were used with 0.5 mL×min^{-1} flow rates: starting at 97/3 (A/B, v/v) and changing to 30/70 (A/B, v/v) in 4 min.

MALDI-ToF mass spectrometry

The MALDI-ToF spectrum was acquired using a 337 nm laser Bruker Autoflex MALDI-ToF mass spectrometer (Bruker) with pulsed ion extraction. The masses were determined in positive ion linear mode. The sample solutions were applied on a ground steel target using the dried droplet technique. First, 0.5 µl of the sample solution (in DMF) was applied on the target. After evaporation of the solvent, 0.5 µl of a solution of dithranol (10 mg/mL) and sodium trifluoroacetate (1 mg/mL) in chloroform was applied on top. Mass calibration was performed with external calibration.

References

(1) Arias, S.; Amini, S.; Horsch, J.; Pretzler, M.; Rompel, A.; Melnyk, I.; Sychev, D.; Fery, A.; Börner, H. G. Toward Artificial Mussel-Glue Proteins: Differentiating Sequence Modules for Adhesion and Switchable Cohesion. *Angewandte Chemie (International ed. in English)* **2020**, *59* (42), 18495–18499. DOI: 10.1002/anie.202008515. Published Online: Aug. 19, 2020.

(2) Matoba, Y.; Kihara, S.; Muraki, Y.; Bando, N.; Yoshitsu, H.; Kuroda, T.; Sakaguchi, M.; Kayama, K.; Tai, H.; Hirota, S.; Ogura, T.; Sugiyama, M. Activation Mechanism of the Streptomyces Tyrosinase Assisted by the Caddie Protein. *Biochemistry* **2017**, *56* (41), 5593–5603. DOI: 10.1021/acs.biochem.7b00635. Published Online: Sep. 27, 2017.

(3) Yang, H.-Y.; Chen, C. W. Extracellular and intracellular polyphenol oxidases cause opposite effects on sensitivity of Streptomyces to phenolics: a case of double-edged sword. *PloS one* **2009**, *4* (10), e7462. DOI: 10.1371/journal.pone.0007462. Published Online: Oct. 14, 2009.

(4) Reinicke, S.; Espeel, P.; Stamenović, M. M.; Du Prez, F. E. One-Pot Double Modification of p(NIPAAm): A Tool for Designing Tailor-Made Multiresponsive Polymers. *ACS macro letters* **2013**, *2* (6), 539–543. DOI: 10.1021/mz4002222. Published Online: Jun. 4, 2013.
